# The CD40-ATP-P2X_*7*_ Receptor Pathway: Cell to Cell Cross-Talk to Promote Inflammation and Programmed Cell Death of Endothelial Cells

**DOI:** 10.3389/fimmu.2019.02958

**Published:** 2019-12-17

**Authors:** Carlos S. Subauste

**Affiliations:** ^1^Division of Infectious Diseases and HIV Medicine, Department of Medicine, Case Western Reserve University, Cleveland, OH, United States; ^2^Department of Pathology, Case Western Reserve University, Cleveland, OH, United States

**Keywords:** CD40, glia, retina, endothelial cell, ATP, cytokine, diabetes

## Abstract

Extracellular adenosine 5′-triphosphate (ATP) functions not only as a neurotransmitter but is also released by non-excitable cells and mediates cell–cell communication involving glia. In pathological conditions, extracellular ATP released by astrocytes may act as a “danger” signal that activates microglia and promotes neuroinflammation. This review summarizes *in vitro* and *in vivo* studies that identified CD40 as a novel trigger of ATP release and purinergic-induced inflammation. The use of transgenic mice with expression of CD40 restricted to retinal Müller glia and a model of diabetic retinopathy (a disease where the CD40 pathway is activated) established that CD40 induces release of ATP in Müller glia and triggers in microglia/macrophages purinergic receptor-dependent inflammatory responses that drive the development of retinopathy. The CD40-ATP-P2X_7_ pathway not only amplifies inflammation but also induces death of retinal endothelial cells, an event key to the development of capillary degeneration and retinal ischemia. Taken together, CD40 expressed in non-hematopoietic cells is sufficient to mediate inflammation and tissue pathology as well as cause death of retinal endothelial cells. This process likely contributes to development of degenerate capillaries, a hallmark of diabetic and ischemic retinopathies. Blockade of signaling pathways downstream of CD40 operative in non-hematopoietic cells may offer a novel means of treating diabetic and ischemic retinopathies.

## Introduction

Glia orchestrate homeostasis in neural tissue through cell-to-cell interactions. Communication among glial subsets and communication between glia and other cells of the nervous system are also important during the development of disorders with an inflammatory component. ATP released by astrocytes appears to cause neuroinflammation by activating pro-inflammatory responses in microglia (1). Retinopathies caused by diabetes and ischemia are driven to a significant extent by chronic inflammation (2–4). The CD40 pathway is activated in these retinopathies and CD40 has emerged as a central mediator of inflammatory responses and pathology in these disorders (5–7). Herein I will review our work that identified CD40 expressed in retinal Müller glia as a trigger for secretion of ATP that in turn engages the P2X_7_ receptor leading to pro-inflammatory cytokine production by monocyte/macrophages/microglia and programmed cell death of retinal endothelial cells (7, 8). Through this process, CD40 present in a non-hematopoietic cell amplifies inflammation and causes tissue pathology.

## CD40

CD40 is a member of the TNF receptor superfamily that is expressed in various hematopoietic and non-hematopoietic cells including antigen-presenting cells (B cells, dendritic cells, and monocyte/macrophages), endothelial cells, epithelial cells, vascular smooth muscle cells, retinal Müller glia, fibroblasts, and neurons (6, 9–13). CD154 (CD40 ligand) is expressed primarily on activated CD4^+^ T cells, platelets, and is also present as a biologically active soluble protein present in plasma (14, 15).

Studies in patients with congenital absence of functional CD154 (Hyper IgM syndrome, X-HIM) provide clinical evidence for the central role of this pathway in adaptive immunity (16). CD40-CD154 interaction promotes dendritic cell maturation inducing licensing of these cells for efficient T cell priming (14, 17, 18). This pathway stimulates IL-12 secretion by dendritic cells that in turn promotes CD4^+^ T cell differentiation into Th1 cells (14, 17, 18). It also supports CD8^+^ cytotoxic T lymphocytes (CTL) development and prevents CTL exhaustion (19). CD40-CD154 interaction promotes pro-inflammatory cytokine production by macrophages and activates effector functions that are central to control of intracellular pathogens (14, 18). Indeed, the most important clinical feature of patients with X-HIM is the increased susceptibility to opportunistic infections normally controlled by cell-mediated immunity (16). The CD40-CD154 pathway is also central for humoral immune responses including B cell proliferation, germinal center formation, antibody production, immunoglobulin class switch, and the generation of B cell memory (14, 17, 18).

In contrast to hematopoietic cells, little is known about the physiologic role of CD40 in non-hematopoietic cells. It has been proposed that CD40 promotes survival of neurons in the brain since old CD40^−/−^ mice (16 months of age) have reduced expression of neurofilament isoforms and exhibit evidence compatible with increased neuronal programmed cell death (TUNEL^+^ neurons) (12). In addition, in developing neural tissue, CD40 promotes axon growth in sympathetic neurons and has effects on dendrite growth that vary depending on the class of neurons: CD40 promotes dendrite growth in hippocampal excitatory neurons while it suppresses dendrite growth in striatal inhibitory neurons (20, 21). It is not known whether CD40 regulates the development and survival of retinal neurons. Moreover, the physiologic function of CD40 expressed in non-hematopoietic compartments in other organs is unclear. This may be explained by the low levels of CD40 expression in these compartments under basal conditions. In contrast, CD40 is upregulated in various inflammatory disorders and, through ligand engagement, CD40 triggers pro-inflammatory responses in endothelial cells, vascular smooth muscle cells and epithelial cells that play a key role in the pathogenesis of various disorders such as inflammatory bowel disease, systemic lupus erythematosus, rheumatoid arthritis, multiple sclerosis, graft rejection, and atherosclerosis (22, 23). These responses include increased protein expression of adhesion molecules, chemokines, metalloproteinases, and tissue factor (22, 23). The effects of CD40 ligation on retinal non-hematopoietic cells are discussed below.

## Diabetic and Other Ischemic Retinopathies

Diabetes mellitus has become one of the most important health problems in the world. It is estimated that there are 422 million patients with diabetes worldwide (World Health Organization; www.who.int/diabetes/global-report). Diabetic retinopathy (DR) is a major complication of diabetes and eventually occurs in ~35% of patients with diabetes (24). In addition, DR is the most common cause of vision loss among working-age adults in developed countries (25). The development of DR appears to be multifactorial and mechanisms such as oxidative stress, increased polyol and hexosamine pathway flux, protein kinase C activation, increased formation of advanced glycation-end products and alterations in systemic and local lipid metabolism have been linked to the development of the disease (26, 27). Ample experimental data indicate that low-grade chronic inflammation also plays an important role in the development of DR (2–4).

The vitreous of patients with DR (28) and retinal endothelial cells from diabetic humans and rodents exhibit increased expression of ICAM-1, an event that promotes adherence of leukocytes to the retinal vasculature (leukostasis) (29, 30). This phenomenon is important since blockade of ICAM-1–CD18 interaction diminishes the development of degenerate capillaries in diabetic mice (31). These structures are a hallmark of early diabetic retinopathy and are formed as a consequence of the death of endothelial cells and pericytes, leading to the transformation of capillaries into collapsed sheaths of collagen/extracellular matrix structures that lack blood flow (32). The ensuing ischemia can promote transition to proliferative DR (PDR) that is characterized by retinal neovascularization. DR is also accompanied by increased expression of TNF-α and IL-1β (33–36). Microglia/macrophages express TNF-α in the diabetic retina (34). TNF-α and IL-1β play a pathogenic role in DR since they contribute to diabetes-induced degeneration of retinal capillaries (37, 38). Inducible nitric oxide synthase (NOS2) is expressed in the retinas of patients with DR and of diabetic rodents (39, 40). Furthermore, diabetic NOS2^−/−^ mice have reduced retinal leukostasis and capillary degeneration (41, 42). CCL2 levels are increased in the vitreous fluid in patients with PDR (43) and in retinas of diabetic rodents (44). This chemokine appears to play a pathogenic role in DR since there is a correlation between CCL2 protein levels in the vitreous with the severity of DR (43).

Ischemic retinopathies including those caused by central retinal artery occlusion, retinal vein occlusion, and retarded retinal vascular development in premature infants are important causes of permanent visual impairment and blindness in adults and children (45–47). Like DR, inflammatory responses including TNF-α, IL-1β, nitro-oxidative stress, and chemokines likely play an important role in the pathogenesis of these diseases (48). Retinal injury induced by ischemia/reperfusion (I/R) is a commonly used animal model of ischemic retinopathy (49). I/R-induced retinopathy is characterized by retinal inflammation, loss of ganglion cells, and development of capillary degeneration (49). I/R of the retina causes upregulation of ICAM-1, TNF-α, IL-1, NOS2, and COX-2 (5, 49–53). These responses are pathogenic since approaches to inhibit them are protective against retinal pathology (50–54).

## CD40 in the Development of Diabetic and I/R-Induced Retinopathies

CD40 is expressed in the retina at the level of endothelial cells, Müller glia (important macroglia in the retina), microglia, ganglion cells, and retinal pigment epithelial cells (5, 6, 55, 56). The levels of CD40 expression are low under basal conditions. However, induction or upregulation of CD40 expression is a feature of inflammatory disorders driven by CD40 (57). Indeed, CD40 mRNA is upregulated in the retina of mice with diabetes and mice subjected to retinal I/R (5, 6). Immunohistochemistry and flow cytometry studies to assess protein expression revealed that CD40 is upregulated in retinal endothelial cells, Müller glia and microglia of diabetic mice (6). Importantly, CD40^−/−^ mice are protected from I/R-induced retinopathy and early diabetic retinopathy (5, 6).

Ligation of CD40 in retinal Müller glia upregulates ICAM-1, CCL2, NOS2 at the protein level and stimulates PGE_2_ production (5, 6, 58). CD40 ligation in retinal endothelial cells upregulates ICAM-1 and CCL2 protein levels (5, 6, 58). Retinal endothelial cells also produce CXCL1 following CD40 stimulation, a response that is markedly potentiated by a low concentration of IL-1β (5).

CD40 is central for the development of retinal inflammation and retinopathy induced by I/R. In contrast to wild-type mice, CD40^−/−^ mice subjected to I/R are protected from upregulation of ICAM-1, CXCL1, NOS2, and COX-2 mRNA levels (5). The reduced expression of NOS2 and COX-2 is explained at least in part by diminished recruitment of NOS2^+^ COX-2^+^ leukocytes into the retina of CD40^−/−^ mice (5). Importantly, the loss of ganglion cells and the development of capillary degeneration are markedly attenuated in ischemic retinas of CD40^−/−^ mice (5). The protection from development of ischemic retinopathy observed in CD40^−/−^ mice is likely explained by diminished leukocyte infiltration and reduced expression of pro-inflammatory molecules since blockade of ICAM-1, NOS2, or COX-2 protect from retinal pathology after ischemia (51, 52, 54). Altogether, CD40 is a central mediator of inflammation and neuro-vascular degeneration after I/R-induced injury of the retina. The model that likely explains these findings is as follows: ischemia-induced activation of CD40 in retinal endothelial cells triggers ICAM-1 and KC/CXCL1 upregulation leading to recruitment of NOS2 and COX-2-expressing leukocytes that would in turn promote neurovascular degeneration in the retina (5). However, it is also possible that Müller glia from ischemic retinas could be a source of increased NOS2 and/or COX-2 expression after activation via CD40.

The upregulation of CD40 and CD154 indicate that this pathway is activated in diabetes. CD40 protein expression in increased in the retina of diabetic mice and in the kidneys of patients with diabetic nephropathy (6, 59). CD40 mRNA levels are upregulated in the retinas of diabetic mice (6). Peripheral blood mononuclear cells from poorly controlled patients with type I diabetes exhibit increased mRNA levels of the functional type I isoform of CD40 (60). It is not known whether changes in micro RNA that control CD40 transcription [i.e., miR-155, miR-424, miR-503 (61, 62)] explain the upregulation of CD40 mRNA. In addition, CD154 protein levels are elevated in the blood from patients with diabetic microangiopathy and mice with diabetes (7, 63, 64). CD154 upregulation is biologically relevant since serum CD154 from diabetics triggers pro-inflammatory responses in endothelial cells and monocytes (63). It is likely that CD154 levels are also increased in the retina because microthrombosis occurs in diabetic retinopathy and activated platelets express CD154 (65).

CD40 is relevant to DR since diabetic CD40^−/−^ mice are protected from upregulation of ICAM-1 in retinal endothelial cells, leukostasis, upregulation of TNF-α, IL-1β, and NOS2 mRNA levels, retinal protein nitration and elevated CCL2 mRNA levels in the retina (6, 7, 58). Importantly, diabetic CD40^−/−^ mice do not develop capillary degeneration (6, 7). Taken together, CD40 is critical for development of various inflammatory responses in the diabetic retina and the development early DR (6, 7).

## CD40 in Müller Glia Recruits Inflammatory Responses in Bystander Microglia/Macrophages

Leukocytes are recognized key players in the development of inflammatory disorders. Indeed, expression of NOS2 or poly(ADP-ribosyl) polymerase 1 (PARP1) in bone marrow cells is necessary for the development of early DR (66). Similarly, CD40 in hematopoietic cells has been deemed a central driver of inflammation. However, studies in mice using bone marrow chimeras revealed that CD40 expressed in non-hematopoietic cells is also required for inflammation (5). Absence of CD40 in the retina inhibits ICAM-1 mRNA upregulation, leukocyte recruitment to the retina and neurovascular degeneration after I/R of the retina (5). Importantly, studies using transgenic mice have established that CD40 expression in a non-hematopoietic cell—Müller glia—is sufficient for development of an inflammatory disorder (7).

Müller glia link with neurons and capillaries, and are central to retina homeostasis (67, 68). Müller glia become dysfunctional and acquire expression of proinflammatory genes in diabetic and other ischemic retinopathies (69–71). The fact that Müller glia express CD40 raised the possibility that CD40 present in these cells may be an important activator of inflammation and retinal injury. Studies in transgenic mice that expressed CD40 restricted to Müller glia demonstrated that, after induction of diabetes, the presence of CD40 in these cells was sufficient for upregulation of ICAM-1, NOS2, TNF-α, IL-1β, CCL2 mRNA levels as well as for development of leukostasis and capillary degeneration (7). This work identified CD40 in Müller glia as a central regulator of inflammation and development of early diabetic retinopathy.

Despite the fact that CD40 in Müller glia from diabetic mice drives TNF-α and IL-1β *in vivo*, work done *in vitro* revealed that human and rodent Müller glia are unable to secrete these pro-inflammatory cytokines in response to CD40 ligation even though these cells react to CD40 stimulation (CCL2 secretion and ICAM-1 protein upregulation) (7). This apparent discrepancy raised the possibility that CD40 in Müller glia acts on bystander microglia/macrophages to promote expression of TNF-α and IL-1β.

Testing whether Müller glia activated by CD40 induce IL-1β and TNF-α production in bystander monocytes/macrophages was done by adding human CD154 to human CD40^+^ Müller glia incubated with CD40^−^ human monocytic cells (to avoid the effects of direct CD40 ligation on these cells), or by adding human CD154 to human CD40-expressing mouse Müller glia incubated with mouse macrophages (human CD154 does not stimulate mouse CD40 expressed in macrophages) (7). While Müller glia and monocyte/macrophages failed to secrete TNF-α and IL-1β in response to CD154, addition of CD154 to the co-culture of these cells triggered TNF-α and IL-1β production (7). The *in vitro* studies have an *in vivo* correlate since diabetic mice that express CD40 restricted to Müller glia upregulate TNF-α protein levels in microglia/macrophages but not in Müller glia while the latter cells upregulate CCL2 protein levels (7). Taken together, these studies revealed that Müller glia activated by CD40 induce pro-inflammatory responses in bystander microglia/macrophages.

## The CD40-ATP-P2X7 Pathway and Inflammatory Responses in Bystander Microglia/Macrophages

ATP functions not only as a neurotransmitter for neurons but can also be secreted by non-excitable cells (72, 73). Moreover, various cell types express P2 purinergic receptors. These receptors are divided into ATP-gated ionotropic P2X receptors and metabotropic, G protein-coupled P2Y receptors (72, 73). The seven subtypes of P2X receptors are ligand-gated channels permeable to Ca^2+^, Na^+^, and K^+^. P2X_7_ receptor is characterized by the ability to form large trans-membrane pores in response to repetitive or prolonged exposure to ATP (72, 73). P2X_7_ receptor is key for IL-1β and TNF-α secretion by microglia/macrophages stimulated with ATP (74, 75). Indeed, secretion of ATP by astrocytes may cause P2X_7_-dependent microglial activation that would drive neuroinflammatory and degenerative disorders (76).

*In vitro* and *in vivo* studies were conducted to determine whether CD40 acts through ATP-P2X_7_ signaling to induce cytokine production in bystander myeloid cells. These studies showed that CD40 is an inducer of ATP release in Müller glia (7). Moreover, purinergic signaling explains TNF-α and IL-1β secretion in bystander monocytes/macrophages incubated with Müller glia activated by CD40. Blockade of the P2X_7_ receptor either by pharmacologic approaches, knockdown of P2X_7_ or the use of macrophages from ${\text{P2X}}_{7}^{-/-}$ mice results in marked inhibition of TNF-α and IL-1β secretion (7). In addition, a purinergic receptor ligand (Bz-ATP) enhances cytokine production by monocytic cells (7).

**Figure 1:**
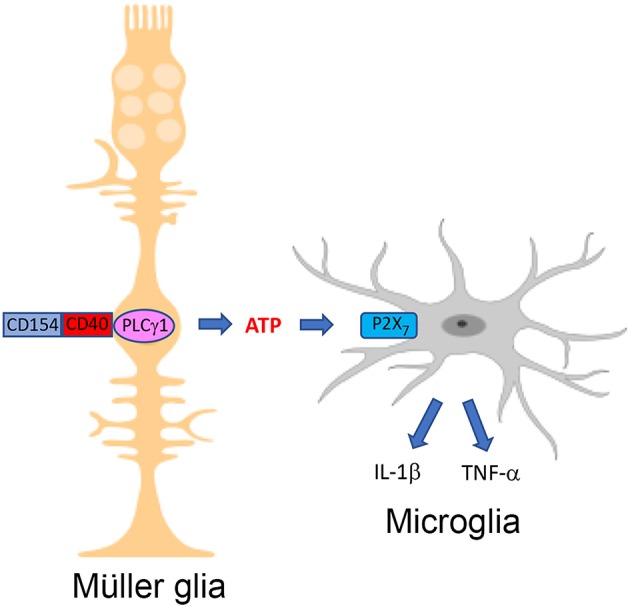
The CD40-ATP-P2X_7_ pathway links cellular responses in Müller glia with the induction of inflammatory responses in bystander microglia/macrophages in DR. Blood levels of CD154 and expression of CD40 in Müller glia are increased in diabetes. CD40 ligation in Müller glia causes PLCγ1 (PLC) activation and secretion of extracellular ATP. P2X_7_ receptor is upregulated in microglia/macrophages in the diabetic retina. ATP binds P2X_7_ receptor leading to secretion of TNF-α and IL-1β. Created in part with BioRender.com.

As described above, studies in diabetic transgenic mice that express CD40 only in Müller glia revealed that TNF-α is expressed in a distinct compartment—microglia/macrophages (7). Moreover, P2X_7_ receptor mRNA levels are enhanced in the retinas of diabetic mice and P2X_7_ receptor protein expression is increased in microglia/macrophages from these animals (7). This is relevant since increased levels of P2X_7_ receptor facilitate the effects of the receptor (77). Mice treated with the P2X_7_ receptor inhibitor BBG as well as ${\text{P2X}}_{7}^{-/-}$ mice are protected from diabetes-induced upregulation of IL-1β and TNF-α mRNA levels (7). The mice are also protected from increased expression of ICAM-1 and NOS2, molecules that are upregulated by IL-1β and TNF-α (78, 79). Taken together, Müller glia activated by CD40 secrete extracellular ATP and drive P2X_7_ receptor-dependent pro-inflammatory cytokine expression in bystander microglia/macrophages *in vitro* and *in vivo* (Figure 1 and Table 1). These findings support a model whereby CD40 engagement in non-hematopoietic cells triggers inflammatory responses not only in these cells (i.e., chemokine and adhesion molecule upregulation) but also amplifies inflammation by enabling bystander myeloid cells to secrete pro-inflammatory cytokines in a manner dependent on ATP-P2X_7_ receptor.

**Table 1:** Components of the CD40-ATP-P2X_7_ pathway in retinopathies.

Müller glia	Important macroglia in the retina that closely communicates with various retinal cells (67, 68)
CD40	Expressed in Müller glia, retinal endothelial cells, microglia/macrophages, and is upregulated in diabetic and I/R-induced retinopathies (5, 6, 55, 56)
CD154	Major ligand of CD40 that is upregulated in plasma and likely in retinal microthrombi in diabetes (7, 63–65)
PLCγ1	Activated by CD40 ligation in Müller glia and triggers release of extracellular ATP (7)
ATP	Secreted by CD40-activated Müller glia and mediates Müller glia—microglia/macrophages and Müller glia—endothelial cell communication (7, 8)
P2X_7_ receptor	ATP receptor upregulated in microglia and retinal endothelial cells in a CD40-dependent manner. Induces pro-inflammatory cytokine secretion by macrophages/microglia and programmed cell death in endothelial cells (7, 8)
TNF-α/IL-1β	Upregulated in diabetic and I/R-induced retinopathies, and linked to development of these retinopathies (33–38, 48–50, 53). CD40 promotes TNF-α/IL-1β upregulation (11)
ICAM-1	Upregulated in endothelial cells in diabetic and I/R-induced retinopathies and linked to development of these retinopathies (3, 29, 30, 49, 54). CD40 promotes ICAM-1 upregulation in retinal endothelial cells (5, 6, 58)
CCL2	Upregulated in diabetic retinopathy and levels of CCL2 are associated with severity of the disease (43, 44). CD40 promotes CCL2 production in endothelial cells and Müller glia (6, 58)
CXCL1	Upregulated in I/R-induced retinopathy (5). CD40 promotes CXCL1 production by endothelial cells (5)
NOS2/COX-2	Upregulated in diabetic and/or I/R-induced retinopathies and linked to development of retinopathies (39–42, 49, 51, 52). CD40 drives NOS2/COX-2 upregulation (5, 6)

Increased intracytoplasmic Ca^2+^ triggers ATP release (80) and CD40 elevates intracytoplasmic Ca^2+^ levels (81, 82). Indeed, BAPTA-AM, a chelator of intracellular Ca^2+^, impairs CD40-mediated ATP release in Müller cells (7). In addition, CD40 ligation in Müller glia causes rapid Tyr783 phosphorylation of phospholipase Cγ1 (PLCγ1) (7), a signaling molecule that increases intracytoplasmic Ca^2+^ (83), and pharmacologic inhibition of PLC impairs ATP release by Müller glia activated by CD40 (7). Thus, CD40 ligation phosphorylates PLCγ1 and CD40 likely functions via PLCγ1 to trigger ATP release in Müller glia.

## CD40 in Müller Glia and Programmed Cell Death of Bystander Retinal Endothelial Cells

Retinal endothelial cells undergo programmed cell death (PCD) in the diabetic retina (32, 84–86). This process would contribute to the development of capillary degeneration, a central feature of early diabetic retinopathy (32). CD40 is necessary for the development of capillary degeneration (6, 7) and yet, ligation of CD40 in endothelial cells does not induce PCD likely because CD40 typically triggers pro-survival signals (87). This raised the possibility of CD40 promoting death of retinal endothelial cells by acting through other cells of the retina. Müller cells were a likely culprit since they encircle retinal endothelial cells.

**Figure 2:**
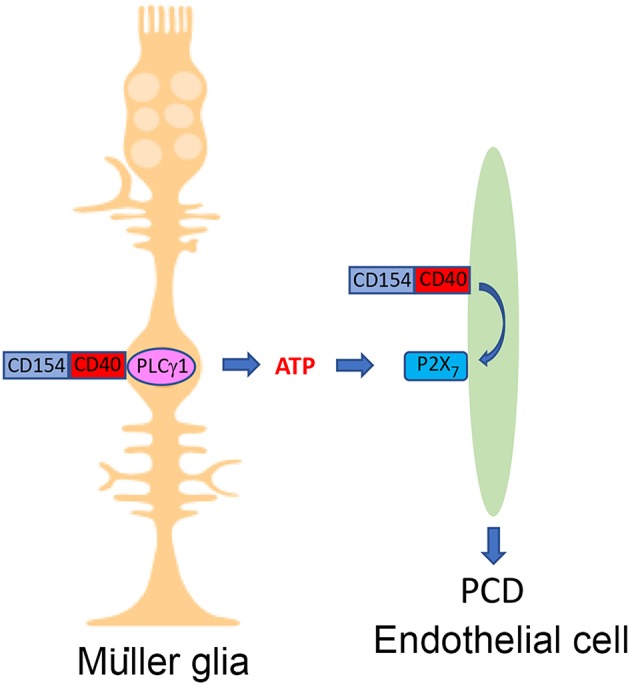
The CD40-ATP-P2X_7_ pathway links cellular responses in Müller glia with programmed cell death of bystander retinal endothelial cells in DR. Blood levels of CD154 and expression of CD40 on Müller glia are increased in diabetes. CD40 ligation in Müller glia causes PLCγ1 (PLC)-dependent secretion of extracellular ATP. CD40 ligation in retinal endothelial cells upregulates P2X_7_ receptor, making these cells susceptible to P2X_7_-induced programmed cell death. Created in part with BioRender.com.

Whereas direct CD40 ligation in retinal endothelial cells does not cause PCD, CD40 stimulation enhances PCD of endothelial cells when they are incubated with CD40^+^ Müller cells (8). This effect is not driven by NOS2, oxidative stress, TNF-α, IL-1β, or Fas ligand (8). As described above, CD40 ligation in Müller glia increases release of ATP. CD40 ligation in retinal endothelial cells upregulates P2X_7_ receptor expression making these cells susceptible to ATP-induced PCD (8). Indeed, pharmacologic inhibition of P2X_7_ receptor prevents PCD of the endothelial cells (8). These results are consistent with the ability of the P2X_7_ to form trans-membrane pores that are permeable to hydrophilic molecules of up to 900 Da (88) and mediate cell death (89, 90). The *in vitro* studies described above have an *in vivo* correlate since retinal P2X_7_ mRNA levels and P2X_7_ receptor expression in retinal endothelial cells are increased in diabetic mice in a CD40-dependent manner (8), CD40 appears to be necessary for PCD of retinal endothelial cells from diabetic mice (8), and CD40 is known to be required for retinal capillary degeneration (6, 7). Taken together, CD40 has a dual role in promoting PCD of retinal endothelial cells: it causes release of extracellular ATP by Müller glia and makes retinal endothelial cells susceptible to P2X_7_-driven PCD (Figure 2 and Table 1). The latter effect may be explained by CD40-driven upregulation of the P2X_7_ receptor in endothelial cells that would overcome the pro-survival signals activated by CD40 ligation. This mechanism may contribute to increased susceptibility to ATP-mediated PCD that appears to occur in diabetes (91). Other potential mechanisms by which CD40 increases susceptibility to P2X_7_ receptor-mediated PCD may include modulation of ATP-gated channel expression, ectoATPase activity, and/or coupling to downstream cell signaling pathways that promote cell death. Finally, while CD40-induced activation of ATP-P2X_7_ receptor signaling mediates PCD of retinal endothelial cells, CD40 may also promote death of these cells and capillary degeneration through mechanisms that include: enhancement of retinal leukostasis, upregulation of NOS2, TNF-α, and IL-1β in the retinas of diabetic mice (6, 7), events linked to PCD of retinal endothelial cells and capillary degeneration (31, 37, 38, 40, 42, 86).

In summary, the studies discussed here discovered the CD40-ATP-P2X_7_ receptor pathway and revealed that this pathway links a macroglia to microglia/macrophages and endothelial cells for the induction of inflammatory responses and endothelial cell death, respectively. By enabling myeloid cells to secrete TNF-α and IL-1β, this process would circumvent the poor capacity of CD40 to directly trigger secretion of these cytokines in non-hematopoietic cells, thus causing amplification of inflammation. These findings may be operative in I/R-induced retinopathy given its similarity to DR. The CD40-ATP-P2X_7_ receptor pathway may also be relevant to neuro-inflammatory and neuro-degenerative brain disorders. For example, astrocytes acquire CD40 expression after incubation with IFN-γ (92), neural tissue injury or in a transgenic mouse model of amyotrophic lateral sclerosis (93), a disease driven by CD40. Thus, the CD40-ATP-P2X_7_ receptor pathway may potentiate pro-inflammatory cytokine production by microglia further driving neuro-inflammation. Finally, this pathway may be functional in other diseases driven by CD40 such as inflammatory bowel disease, atherosclerosis and lupus nephritis. CD40 present in non-hematopoietic cells of the intestine, blood vessels and kidney may induce release of ATP that would bind purinergic receptors present in infiltrating myeloid cells.

The existence of the CD40-ATP-P2X_7_ receptor pathway may have therapeutic implications. Pre-clinical data revealed that administration of anti-CD154 mAb to inhibit CD40-CD154 signaling effectively controlled various inflammatory and neurodegenerative disorders (22, 23). Unfortunately, anti-CD154 mAbs caused thromboembolic complications in humans that are unrelated to inhibition of CD40 (94). Targeting signaling pathways downstream of CD40 may represent an alternative approach to treat CD40-driven diseases. CD40 functions by recruiting TNF Receptor Associated Factors (TRAF) to its TRAF2,3 or TRAF6 binding sites (95). Blockade of CD40-TRAF2,3 signaling markedly impairs pro-inflammatory responses in non-hematopoietic cells (58, 96). Blocking this signaling pathway may also inhibit pro-inflammatory responses in neighboring myeloid cells. Pharmacologic approaches to inhibit CD40-TRAF2,3 signaling (cell penetrating CD40-TRAF2,3 blocking peptide or small molecule CD40-TRAF2,3 inhibitor) may prove an effective approach to treat diabetic and ischemic retinopathies, and potentially other CD40-driven inflammatory disorders. Given that TRAF6 is critical for dendritic cell maturation and development (96, 97), CD40-mediated IL-12 production by dendritic cells (98) and induction of antimicrobial effector mechanisms in macrophages (99, 100), pharmacologic inhibition of CD40-TRAF2,3 signaling would minimize the risk of opportunistic infections by leaving CD40-TRAF6 signaling intact.

## Author Contributions

The author confirms being the sole contributor of this work and has approved it for publication.

### Conflict of Interest

The author declares that the research was conducted in the absence of any commercial or financial relationships that could be construed as a potential conflict of interest.
